# Rac1 overexpression promotes Treg-derived cytokines to mediate choroidal neovascularization in wet age-related macular degeneration

**DOI:** 10.1590/1414-431X2024e14187

**Published:** 2025-03-03

**Authors:** Juanjuan Li, Yuling Ren, Hua Li, Zhikun Zheng

**Affiliations:** 1 Affiliated Hospital of Yunnan University (Second People's Hospital of Yunnan Province, Yunnan Eye Hospital), Kunming, Yunnan, China

**Keywords:** Age-related macular degeneration (AMD), Regulatory T cells (Tregs), Choroidal neovascularization, Angiogenesis, Rac1

## Abstract

Age-related macular degeneration (AMD), particularly the wet form characterized by choroidal neovascularization, is a leading cause of vision loss. Dysregulation of regulatory T cells (Tregs), key modulators of inflammatory responses, may contribute to wet AMD pathogenesis. This study explored the involvement of Tregs and the Rac1 signaling pathway in modulating Treg-derived cytokine expression and their role in choroidal neovascularization during wet AMD progression. Peripheral blood samples from healthy controls, dry AMD patients, and wet AMD patients were collected. An *in vitro* transmembrane co-culture system of Tregs and human choroidal endothelial cells (HCECs) was employed to investigate the impact of Tregs (with or without Rac1 silencing) on the angiogenic phenotype of HCECs. A mouse model of AMD was established to evaluate the effects of a Rac1 inhibitor and IL-10/TGF-β neutralization on Tregs and choroidal neovascularization. An increased Treg percentage in the CD4+ T lymphocyte population was found in the peripheral blood samples of wet AMD patients. Tregs from wet AMD patients showed an increased expression of Rac1 and an elevated production of IL-10 and TGF-β1. Rac1 silencing suppressed Treg stability and differentiation, and impaired the pro-angiogenic effect of Tregs on HCECs. In the animal model of AMD, the administration of a Rac1 inhibitor or neutralizing antibodies against IL-10/TGF-β1 reduced Treg abundance and attenuated choroidal neovascularization. Rac1 upregulation in Tregs promoted IL-10 and TGF-β1 production to mediate choroidal neovascularization in wet AMD. Targeting Rac1 and Treg-derived IL-10/TGF-β1 production in Tregs may serve as a strategy to ameliorate AMD progression.

## Introduction

With the aging of the world population, the incidence of age-related macular degeneration (AMD) and other common ophthalmic diseases has increased year by year, which has become one of the main causes of vision decline and blindness among the elderly worldwide (1). According to epidemiological data, the number of people with moderate visual impairment in China increased by 133.67% from 1990 to 2019, and the incidence of severe visual impairment increased by 147.14% (2). The main reason for the prevalence of visual impairment in the elderly population is the increasing incidence of cataracts and AMD (2,3). The loss of visual capacity due to AMD or cataracts could seriously undermine the quality of life for the elderly.

According to pathological features, AMD can be categorized as dry AMD and wet AMD (4). Dry AMD progresses slowly, accounting for 90% of all cases of AMD (5). Wet AMD is characterized by choroidal neovascularization and accounts for only 10% of AMD cases. However, the disease progresses rapidly and the prognosis is poor (6). Due to the complex etiology of AMD, there are numerous hypotheses regarding the pathogenesis of AMD, including immune inflammation, extracellular matrix changes, retinal pigment epithelium dysfunction, oxidative stress, and angiogenic disorders (6,7).

The phenotypic and functional differentiation of different immune cells in pathological processes can contribute to the development of AMD. In previous studies, macrophage recruitment and the pro-angiogenic polarization of resident choroidal macrophages have been implicated in the choroidal neovascularization of AMD (8,9). Interleukin (IL)-1β and tumor necrosis factor (TNF)-α secreted by macrophages can promote angiogenesis in choroidal neovascular membranes by stimulating the production of vascular endothelial growth factors (VEGFs) (10,11). Regulatory T cells (Tregs) are a subset of CD4+T cells with immunosuppressive function, which exert inhibitory effects on a variety of immune cells to maintain peripheral immune tolerance (12,13). Tregs produce IL-10 and transforming growth factor (TGF)-β as inhibitory cytokines to dampen the activation of immune cells or directly suppress effector T cells by competitively consuming IL-2 (14,15). IL-10 is an important anti-inflammatory factor which promotes neovascularization under different pathological conditions (16,17). Under hypoxia, an increased expression of IL-10 in retinal tissue can stimulate the production of VEGFs from macrophages, increasing the risk of retina angiogenesis (18). In a mouse model of AMD, it was reported that the expression of TGF-β levels was significantly increased (19). Furthermore, there is evidence to suggest that the expression of Treg master regulator FoxP3 was elevated in the retinal tissues of AMD patients (20). However, how Treg functions are dysregulated in wet AMD patients and whether Treg-derived IL-10 and TGF-β contribute to the choroidal neovascularization in AMD progression remain to be explored. Our published work revealed that silencing of Rac1 expression via RNA interference inhibits retinal neovascularization in rats (21). In the current study, we investigated the interplay between Rac1 and Tregs in the pathogenesis of choroidal neovascularization that had not been investigated.

## Material and Methods

### Clinical samples

Peripheral venous blood samples were collected from healthy controls, dry AMD patients, and wet AMD patients (n=20 in each category) at the Affiliated Hospital of Yunnan University (China). The samples were stored at 4°C and subjected to cell isolation and analyses within 24 h. All of the subjects signed a written informed consent. The use of human samples was approved by the Medical Research Ethics Committee of the Affiliated Hospital of Yunnan University (Approval number: 2022144).

### Primary cell isolation

Total lymphocytes from blood samples were purified using the EasySep Direct Human Total Lymphocyte Isolation Kit (Stem Cell Technologies, USA). The CD4+ T cell population was isolated using the EasySep Human CD4+ T Cell Isolation Kit (Stem Cell Technologies). The primary CD4+ lymphocytes were cultivated in RPMI-1640 medium containing 10% fetal calf serum (FCS), 50 μg/mL gentamicin, 50 μM 2-mercaptoethanol, and 5 ng/mL recombinant human IL-2 (Procell, China). To isolate Tregs from peripheral blood samples, a CD4+CD25+ Treg Isolation Kit, human (Miltenyi Biotec, USA) was used based on the supplier's instructions. The purified Treg cells were expanded using a Human Treg Expansion Kit (Miltenyi Biotec).

### Flow cytometry

For cell surface staining, cells were washed in PBS supplemented with 2% FBS and blocked in anti-FcR monoclonal antibody (Cat# 16-0161-82, 5 μg/mL, eBioscience, USA) at 4°C for 15 min. Afterward, the surface staining of CD4 and CD25 was conducted using the fluorophore-conjugated antibodies (PB-αCD4 Cat# 48-0042-82, 0.5 μg/mL; PE-αCD25 Cat# 12-0259-42, 0.2 μg/mL; eBioscience) at 4°C for 15 min. For the intracellular FoxP3 staining, cells were fixed and permeabilized using the FoxP3 intracellular staining kit (Cat# 00-5523-00, eBioscience) before incubation with anti-FoxP3 antibody (APC-αFoxP3 Cat# 17-5773-82, 2.5 μg/mL, eBioscience) for 30 min at 4°C. After rinsing with the permeabilizing buffer, the stained cells were re-suspended in PBS and analyzed on an LSRII flow cytometer (BD Biosciences, USA).

### ELISA

The quantification of IL-10 and TGF-β1 in blood samples or cell culture supernatants were conducted using commercial ELISA kits (Sangon, China). In brief, assay plates were coated with the capture antibody at 4°C overnight. Then 100 μL of samples or standards were added into each well of the coated plate for a 2-h incubation at room temperature. After rinsing, 100 μL of biotin-conjugated detection antibody was added for a 1-h incubation, followed by labeling with 50 μL of streptavidin-HRP. Next, 100 μL of a chemiluminescent detection reagent was applied for signal development for 30 min at ambient temperature, and the absorbance values of the samples and standards were measured at 450 nm using a microplate reader (Synergy H1, BioTeck, USA).

### Cell culture

Human choroidal endothelial cells (HCECs) were procured from Zeye Biotech (ZY-008H, China). The cells were maintained in McCoy's medium (Procell) with 10% FBS (Sigma, China) and 1% penicillin/streptomycin (Procell) under the conditions of 37°C and 5% CO_2_. For the co-culture experiment of Tregs and HCECs, HCECs were plated together with Tregs at the ratio of 1:0.5 onto a Transwell co-culture system using 0.4 μm Nunc polycarbonate cell culture inserts (Thermo Fisher Scientific, USA) for 48 h, which allows the exchange of materials in the absence of cell contact.

### RT-qPCR

RNA purification from plasma and cell samples was conducted using TRIzol reagent (Qiagen, China), and reverse transcription was performed using the PrimeScript™ RT Reagent Kit (Takara Biotechnology, Japan). The resulting DNA was quantified in a 7500 Real Time PCR System (Applied Biosystems, USA) using SYBR Green qPCR Master Mix (MedChemExpress, China). The 2^-ΔΔCT^ method was adopted for the relative gene expression analysis, with β-actin as the reference gene.

### Western blot

Protein sample isolation was conducted using the RIPA lysis buffer (Beyotime, China) supplemented with protease inhibitor and PMSF (ThermoFisher Scientific). Protein concentration was quantified using a BCA protein assay kit (Beyotime). The following primary antibodies (Abcam, UK) were used for the detection of target proteins: anti-beta actin antibody (Cat# ab8227, 0.25 μg/mL), anti-RAC1 antibody (Cat# ab155938, 0.5 μg/mL), anti-VEGFA antibody (Cat# ab46154, 0.5 μg/mL), and anti-Ang2 antibody (Cat# ab155106, 0.5 μg/mL). The HRP-linked detection antibody (Cat# ab6721, 0.1 μg/mL) was used for the labeling of primary antibodies, which was followed by the signal development using the ECL chemiluminescent solution (Beyotime). The densities of protein bands were analyzed by Image J Software Version 1.53t (NIH, USA).

### Gene silencing

The lentivirus vector containing control shRNA (pLKO.1-puro-sh-Ctrl) and the vector carrying shRNA targeting RAC1 (pLKO.1-puro-sh-RAC1) were synthesized by GenePhama (China). The pSPAX2/pMD2.g (packaging vectors, Addgene, USA) and the lentiviral vector were co-transfected into HEK293 cells using Lipofectamine 3000 (Invitrogen, China) to produce the control and RAC1 silencing lentivirus. Forty-eight hours after the transfection, the supernatant was collected and used to transduce Treg cells in the presence of polybrene (8 μg/mL (MedChemExpress). Cells were used for further experiments 48 h after viral transduction.

### Tube formation assay

The *in vitro* angiogenesis assay kit (ab204726; Abcam) was employed to examine the angiogenic potential. A 96-well assay plate was coated with 50 μL of extracellular matrix (ECM) solution at 37°C for 20 min. HCECs (2.0×10^4^ cells per well) were inoculated onto each well and incubated for 18 h. The tube formation was observed under an Olympus IX81 Inverted Microscope (Olympus, Japan).

### Transwell invasion assay

Cell invasion ability was examined in a 12-mm Transwell chamber with an 8.0-µm pore insert, which was coated with Matrigel (BD Biosciences) at 37°C for 30 min. HCECs (2×10^5^ cells per well) were seeded onto the upper chamber in serum-free medium, and the lower chamber was loaded with complete culture medium. After 24 h, the invading cells on the membrane were fixed and stained with 0.25% crystal violet (Sigma) for 20 min. Cells were photographed and counted under an Olympus IX81 Inverted Microscope (Olympus).

### *In vivo* experiment

BABL/c male mice (8-week-old) were purchased from Weitong Lihua Experimental Animal Technology Co., Ltd. (China) and raised in a standard pathogen-free facility, with free access to food and drinking water. Laser-induced choroidal neovascularization was established in mice as the animal model of AMD. The mice were anesthetized by intraperitoneal (*ip*) injection of acepromazine maleate at 3 mg/kg. A drop of tropicamide (Santen Pharm, China) was applied in each eye to dilate the pupils. The mice were placed perpendicular to the laser beam and the laser beam was focused on the eye fundus. The laser induction was performed using the ReGenTM S1 Class-4 Therapy Laser (ColdLasers, USA), and the power was calibrated to 0.350 W and the exposure time was set to 0.050 s. Afterward, the mice were placed on a warming pad until recovery from anesthesia. The control group of mice were subjected to anesthesia and tropicamide, without laser induction. For the pharmaceutical intervention, mice (n=6 animals in each group) were *ip* injected with RAC1 inhibitor (1A-116, 3 mg/kg, MedChemExpress), TGF beta-1,2,3 Monoclonal Antibody (1D11) (MA5-23795, 1 mg/kg, Invitrogen), or IL-10 Monoclonal Antibody (JES5-2A5, 1 mg/kg, Invitrogen) twice per week. After 14 days, ocular venous blood samples were collected, and the mice were euthanized by cervical dislocation for eye tissue collection. The animal protocols were approved by the Experimental Animal Use Ethics Committee of Yunnan University Animal Research and Resource Center. The number of animals per group (n=6) was determined by power analysis based on previous studies using similar models to detect a minimum biologically relevant effect size of 25% change in neovascularization area with 80% power and α=0.05. The animals were randomized into treatment groups using a random number generator in Microsoft Excel. The mice were assigned a random number, then sorted in ascending order. The first 6 mice were assigned to the control group, the next 6 to the RAC1 inhibitor group, the following 6 to the TGF-beta antibody group, and the last 6 to the IL-10 antibody group. This ensured random and unbiased allocation of animals to the different experimental groups.

### Immunofluorescence staining

The staining was performed in 5-μm sections of formalin-fixed paraffin-embedded (FFPE) tissues. After the initial de-paraffinization and re-hydration, the antigen retrieval was conducted by heating the sections in the citrate antigen recovery solution (Zeye Biotech, China) at 95°C for 15 min, and peroxidase was inactivated in 3% hydrogen peroxide for 15 min. After rinsing and blocking for 1 h in 5% normal goat serum, primary antibody against CD34 (Cat#: ab81289, 2 μg/mL, Abcam) was applied to label the sections overnight. The sections were further labeled with goat anti-rabbit IgG H&L (Alexa Fluor^®^ 488) (Cat#: ab150077, 0.5 μg/mL, Abcam) for 60 min at room temperature. The samples were observed under an Olympus IX81 Inverted Microscope (Olympus). Microvascular density (MVD) was quantified using ImageJ software (Version 1.53t, Wayne Rasband, National Institutes of Health, USA) by converting images to grayscale, setting a threshold to identify CD34+ staining, and measuring the area of positive staining through the “Analyze Particles” function. The data are reported as the relative CD34+ region present in each field.

### Statistical analysis

Data analysis was conducted using SPSS software version 26.0 (IBM, USA). Data are reported as means±SD. Student's *t*-test was adopted for comparing continuous variables between two conditions. Multiple comparisons were performed by one-way ANOVA. P<0.05 was accepted to indicate statistical significance.

## Results

### Increased Treg percentage in CD4+ T lymphocyte population of wet AMD patients

**Figure 1:**
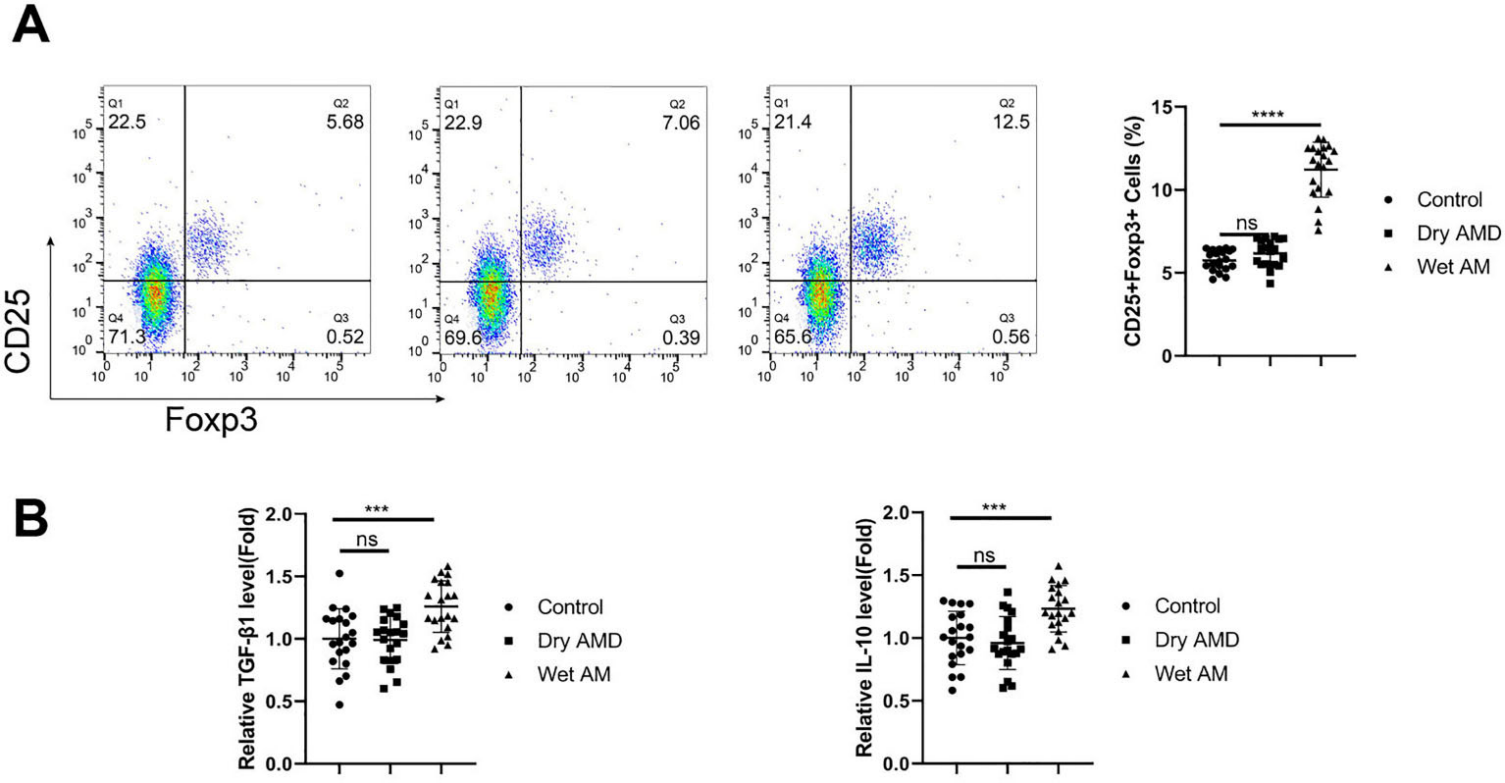
Increased Treg percentage in CD4+ T lymphocyte population of wet age-related macular degeneration (AMD) patients. **A**, Flow cytometry analysis of CD25+FoxP3+ Treg percentage within the CD4+ T cell population of peripheral blood samples from healthy controls, dry AMD patients, and wet AMD patients (n=20 samples per group). **B**, ELISA analysis of Treg-related cytokines (transforming growth factor (TGF)-β1 and interleukin (IL)-10) in the blood samples from each group. Data are reported as means±SD; n=20 samples per group. ***P<0.001; ****P<0.0001; ns: non-significant (ANOVA).

A significant increase was found in the CD25+FoxP3+ Treg percentage within the CD4+ T cell population from wet AMD samples (Figure 1A, P<0.0001). The analysis of Treg-related cytokines (TGF-β1 and IL-10) also revealed increased levels in blood samples of wet AMD patients compared to the other samples (Figure 1B, P<0.001).

### Tregs from wet AMD patients showed increased expression of FoxP3 and Rac1

**Figure 2:**
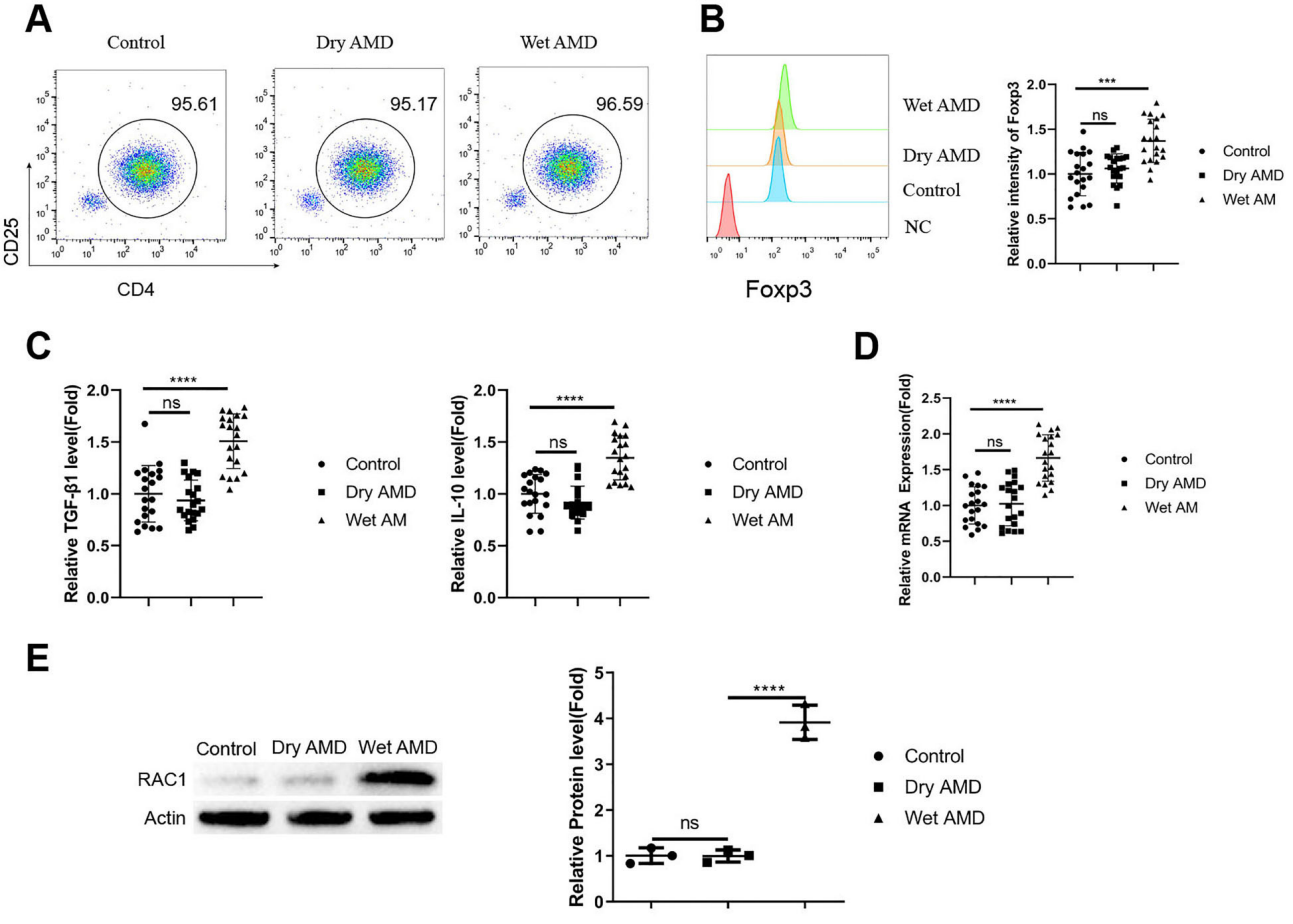
Tregs from wet age-related macular degeneration (AMD) patients showed increased expression of FoxP3 and Rac1. **A**, Purity of CD4+CD25+ Tregs isolated from blood samples of healthy controls, dry AMD patients, and wet AMD patients. **B**, Intracellular FoxP3 staining in isolated Tregs. **C**, ELISA analysis of transforming growth factor (TGF)-β1 and interleukin (IL)-10 levels in the isolated Tregs. **D**, RT-qPCR analysis of Rac1 expression in the isolated Tregs from different samples. **E**, Western blot analysis of Rac1 protein levels in the isolated Tregs. n=20 samples per group in **A**-**D**. n=3 independent samples per group in **E**. Data are reported as means±SD. ***P<0.001; ****P<0.0001; ns: non-significant (ANOVA).

CD4+CD25+ Tregs were purified from the blood samples using magnetic activated cell sorting, and the purified cell populations showed similar purity across the samples from healthy controls, dry AMD patients, and wet AMD patients (Figure 2A). Intracellular FoxP3 staining indicated an increased expression level of FoxP3 in Tregs from wet AMD patients compared to the other two groups (Figure 2B, P<0.001). ELISA analysis demonstrated that there were also increased levels of TGF-β1 and IL-10 in Tregs from wet AMD patients (Figure 2C, P<0.0001). We further examined the expression of Rac1 in Tregs from different samples. RT-qPCR and Western blot showed that both the mRNA and protein levels of Rac1 were elevated in Tregs from wet AMD patients (Figure 2D and E, P<0.0001).

### Rac1 silencing suppressed Treg stability and differentiation

**Figure 3:**
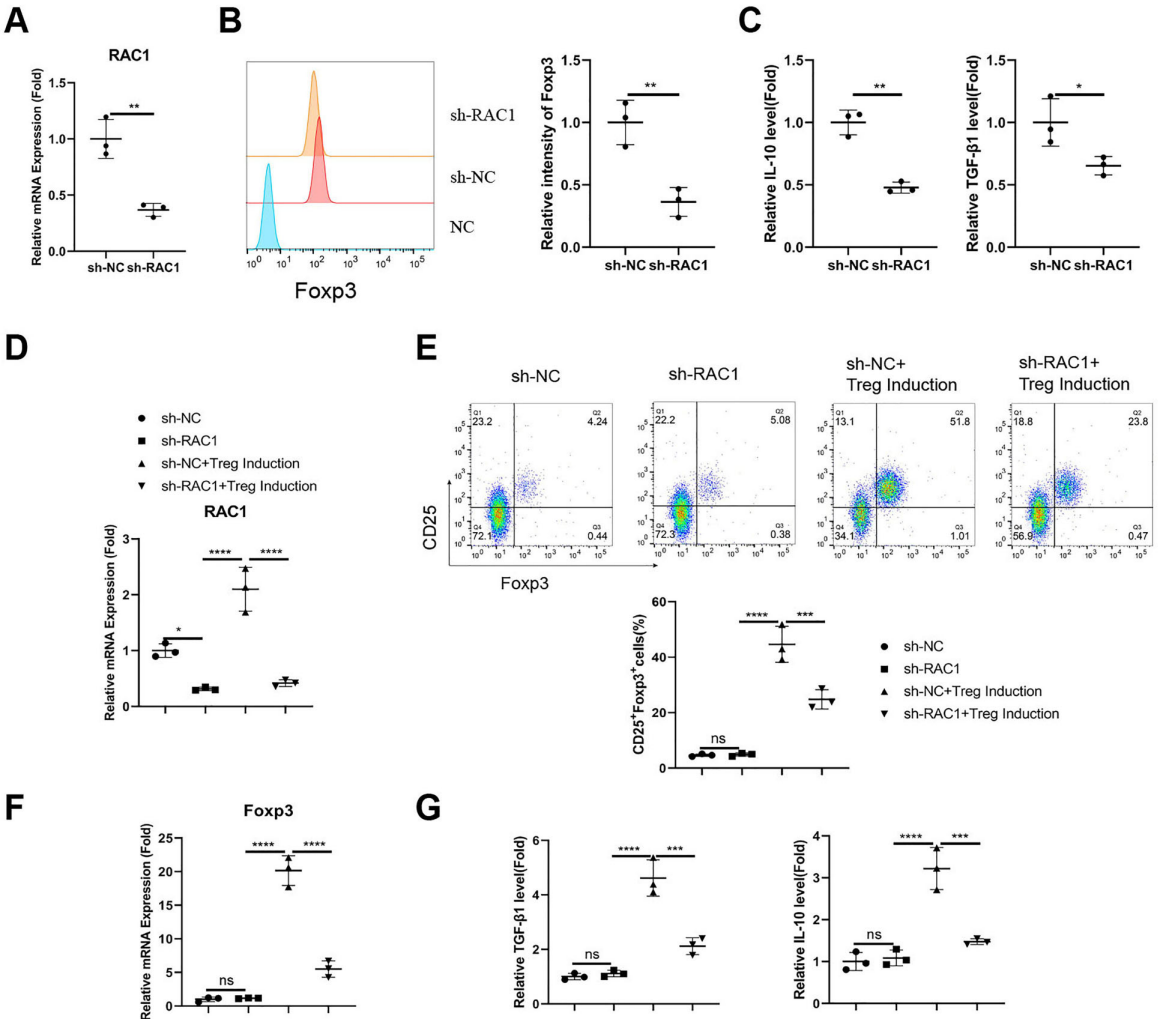
Rac1 silencing suppressed stability and impaired Treg differentiation. Tregs from wet age-related macular degeneration (AMD) patients were infected with a lentivirus carrying a negative control sh-RNA (sh-NC) or an sh-RNA targeting Rac1 (sh-Rac1). **A**, mRNA expression levels of Rac1 in Tregs with or without Rac1 silencing. **B**, Intracellular staining of FoxP3 in Tregs with or without Rac1 silencing. **C**, ELISA analysis of interleukin (IL)-10 and transforming growth factor (TGF-β1) in Tregs with or without Rac1 silencing. **D**, RT-qPCR analysis of Rac1 expression in CD4+CD25- T cells infected with sh-NC or sh-Rac1 lentivrius, with or without Treg differentiation induction. **E**, Analysis of the CD25+FoxP3+ Treg population in CD4+CD25- T cells carrying sh-NC or sh-Rac1, with or without *in vitro* Treg differentiation. **F**, RT-qPCR analysis of Foxp3 expression in CD4+CD25- T cells infected with sh-NC or sh-Rac1 lentivrius, with or without Treg differentiation induction. **G**, ELISA analysis of TGF-β1 and IL-10 levels in CD4+CD25- T cells infected with sh-NC or sh-Rac1 lentivrius, with or without Treg differentiation induction. Data are reported as means±SD; n=3 independent experiments. *P<0.05; **P<0.01; ***P<0.001; ****P<0.0001; ns: non-significant (ANOVA).

We next attempted to examine the functional role of Rac1 in Tregs from wet AMD patients. Tregs were infected with lentivirus carrying a control sh-RNA (sh-NC) or an sh-RNA targeting Rac1 (sh-Rac1). The delivery of sh-Rac1 significantly suppressed the mRNA expression of Rac1 in Tregs (Figure 3A, P<0.01). Upon Rac1 silencing, the expression level of FoxP3 was reduced in Tregs, as revealed by the intracellular staining (Figure 3B, P<0.01). The secretion of TGF-β1 and IL-10 was also decreased upon Rac1 knockdown (Figure 3C, P<0.05 and P<0.01). Next, we silenced Rac1 in the CD4+CD25- T cell population and studied the role of Rac1 in Treg differentiation *in vitro* (Figure 3D, P<0.05 and P<0.0001). Compared to the sh-NC (negative control), silencing of Rac1 significantly suppressed Treg differentiation (Figure 3E, P<0.001), curtailed the induction of FoxP3 expression (Figure 3F, P<0.0001), and attenuated the production of TGF-β1 and IL-10 (Figure 3G, P<0.001) after Treg differentiation induction. Therefore, Rac1 was required to maintain Treg stability and support Treg differentiation.

### Tregs from wet AMD patients showed the strongest effect on the invasion and angiogenesis of HCECs

**Figure 4:**
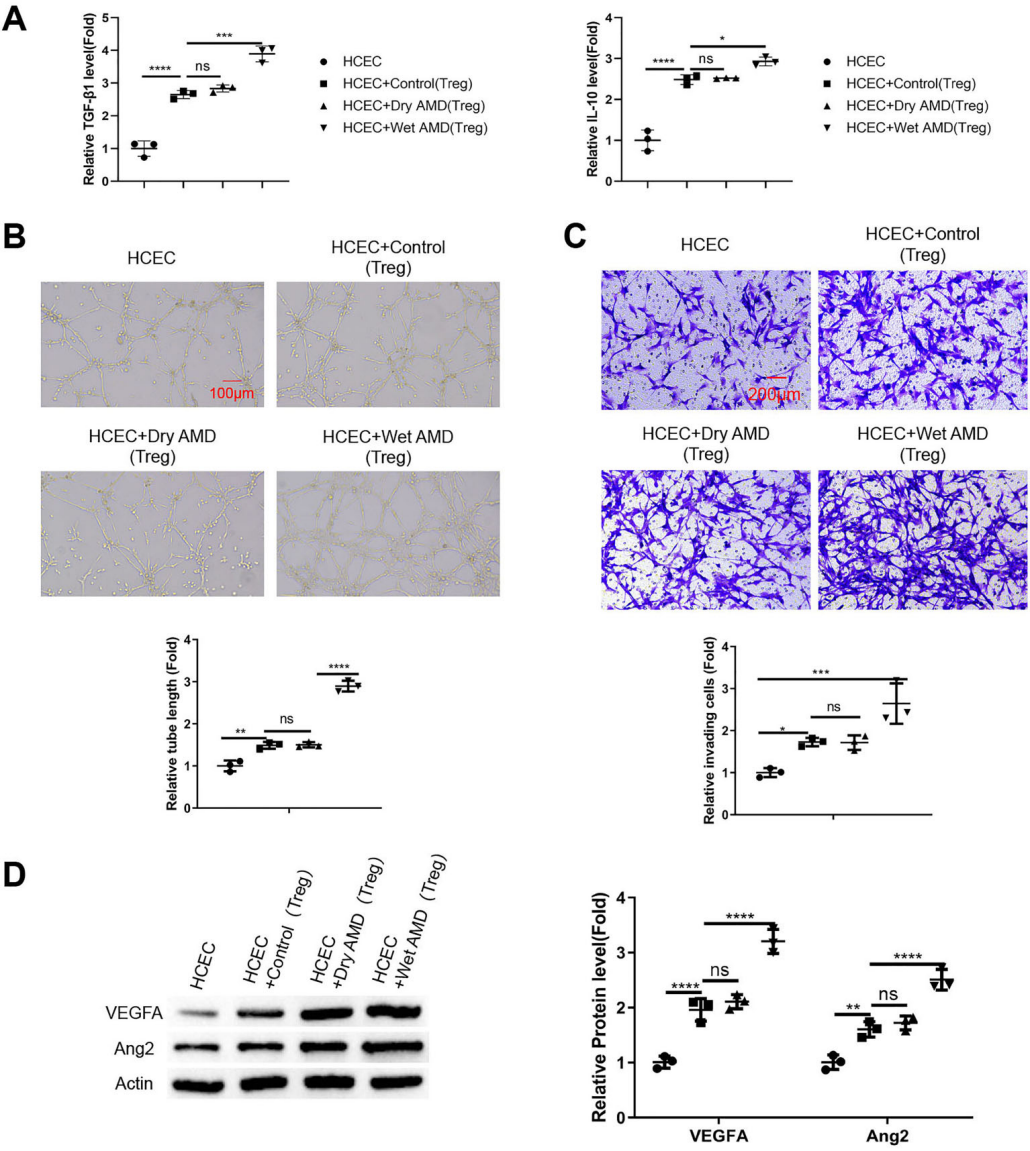
Tregs from wet age-related macular degeneration (AMD) patients exhibited the strongest effects on the invasion and angiogenesis of human choroidal endothelial cells (HCECs). An *in vitro* transmembrane co-culture system was established with Tregs from different groups co-cultured with HCECs. **A**, Levels of transforming growth factor (TGF)-β1 and interleukin (IL)-10 in the media of different co-culture groups were detected by ELISA. **B**, *In vitro* tube formation assay performed with HCECs after co-culture with different groups of Tregs (scale bar 100 μm). **C**, Transwell invasion assay performed with HCECs after co-culture with different groups of Tregs (scale bar 200 μm). **D**, Western blot analysis of the pro-angiogenic factors (VEGFA and Ang2) in HCECs upon co-culture with different groups of Tregs. Data are reported as means±SD; n=3 independent experiments. *P<0.05; **P<0.01; ***P<0.001; ****P<0.0001; ns: non-significant (ANOVA).

An *in vitro* transmembrane co-culture system of Tregs from different groups and HCECs was established to study the impact of Tregs on the phenotype of HCECs. As expected, the presence of Tregs increased the concentration of TGF-β1 and IL-10 in the medium of the co-culture system, and the levels of these cytokines increased to the highest levels in the HCEC+wet AMD Treg group (Figure 4A, P<0.05, P<0.001, and P<0.0001). The *in vitro* tube formation assay demonstrated that the presence of wet AMD Tregs promoted the angiogenic potential of HCECs to the greatest extent (Figure 4B, P<0.01 and P<0.0001). Further, wet AMD Tregs also exerted the strongest effect in enhancing the cell invasion of HCECs in the Transwell invasion assay (Figure 4C, P<0.05 and P<0.001). Furthermore, there was an increased expression of pro-angiogenic factors (VEGFA and Ang2) in HCECs upon their co-culture with Tregs, with Tregs from wet AMD patients showing the most potent effect (Figure 4D, P<0.01 and P<0.0001).

### Rac1 silencing in Tregs from wet AMD patients attenuated the pro-angiogenic effect

**Figure 5:**
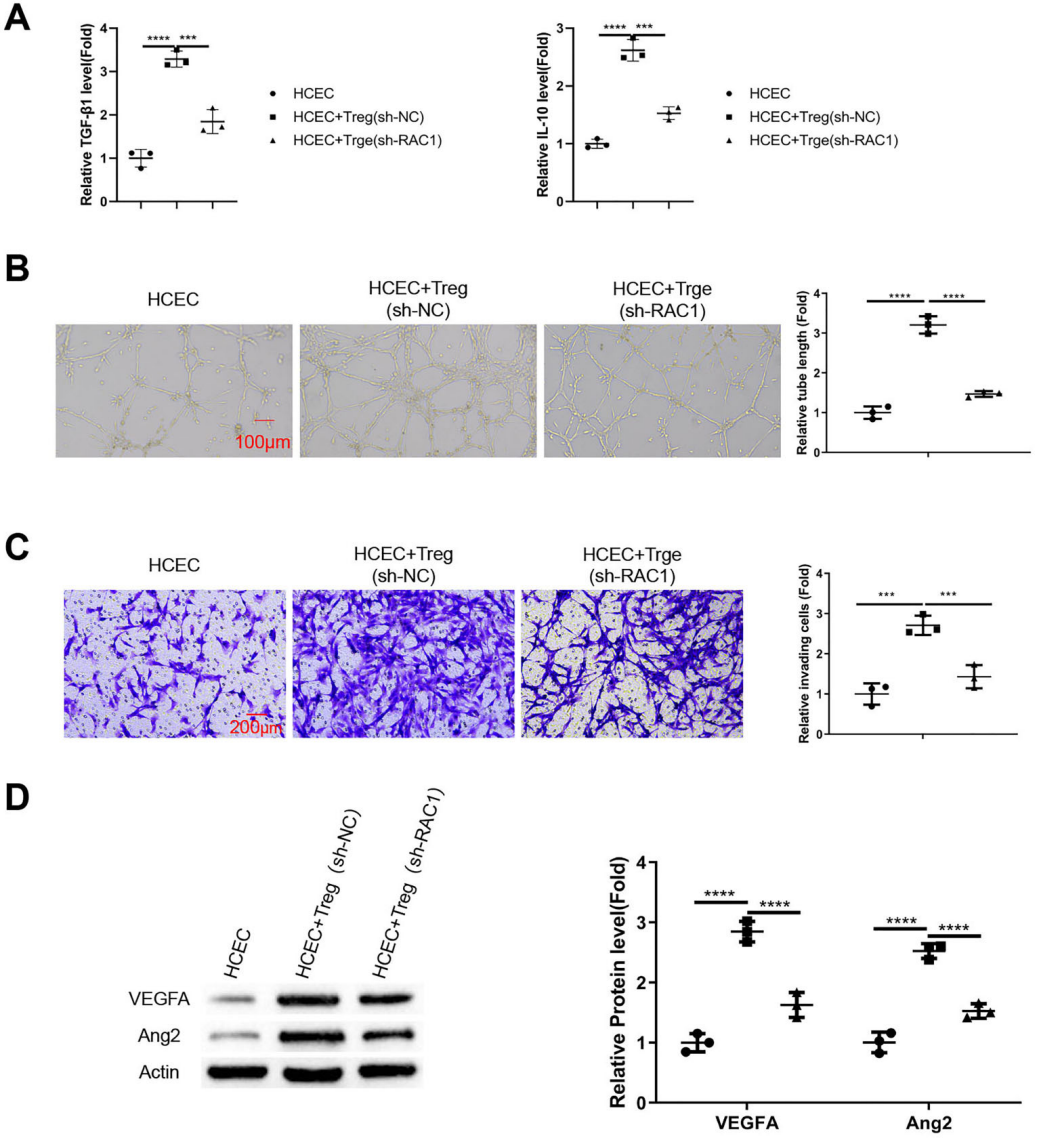
Rac1 silencing in Tregs from wet age-related macular degeneration (AMD) patients attenuated their pro-angiogenic effect. Tregs from wet AMD patients were infected with a lentivirus carrying a negative control sh-RNA (sh-NC) or an sh-RNA targeting Rac1 (sh-Rac1). An *in vitro* transmembrane co-culture system was established with Tregs (with sh-NC or sh-Rac1) co-cultured with human choroidal endothelial cells (HCECs). **A**, ELISA analysis of transforming growth factor (TGF)-β1 and interleukin (IL)-10 levels in the media of different co-culture groups. **B**, *In vitro* tube formation assay performed with HCECs after co-culture with Tregs treated with sh-NC or sh-Rac1 (scale bar 100 μm). **C**, Transwell invasion assay performed with HCECs after co-culture with Tregs treated with sh-NC or sh-Rac1 (scale bar 200 μm). **D**, Western blot analysis of the pro-angiogenic factors (VEGFA and Ang2) in HCECs upon co-culture with Tregs treated with sh-NC or sh-Rac1. Data are reported as means±SD; n=3 independent experiments. ***P<0.001; ****P<0.0001; ns: non-significant (ANOVA).

We next applied Tregs from wet AMD patients with or without Rac1 knockdown in the co-culture system with HCECs. Rac1 silencing reduced the levels of TGF-β1 and IL-10 in the medium of the co-culture system (Figure 5A, P<0.001 and P<0.0001). Compared to the Tregs carrying sh-NC, the pro-angiogenic effect of Tregs with Rac1 knockdown was significantly impaired (Figure 5B, P<0.0001). A similar effect was observed in the Transwell invasion assay (Figure 5C, P<0.01 and P<0.001). Consistently, the expression levels of pro-angiogenic factors (VEGFA and Ang2) in HCECs were reduced upon their co-culture with Tregs with Rac1 silencing (Figure 5D, P<0.0001).

### Rac1 activity mediated Treg functions to support choroidal neovascularization in the mouse model of AMD

**Figure 6:**
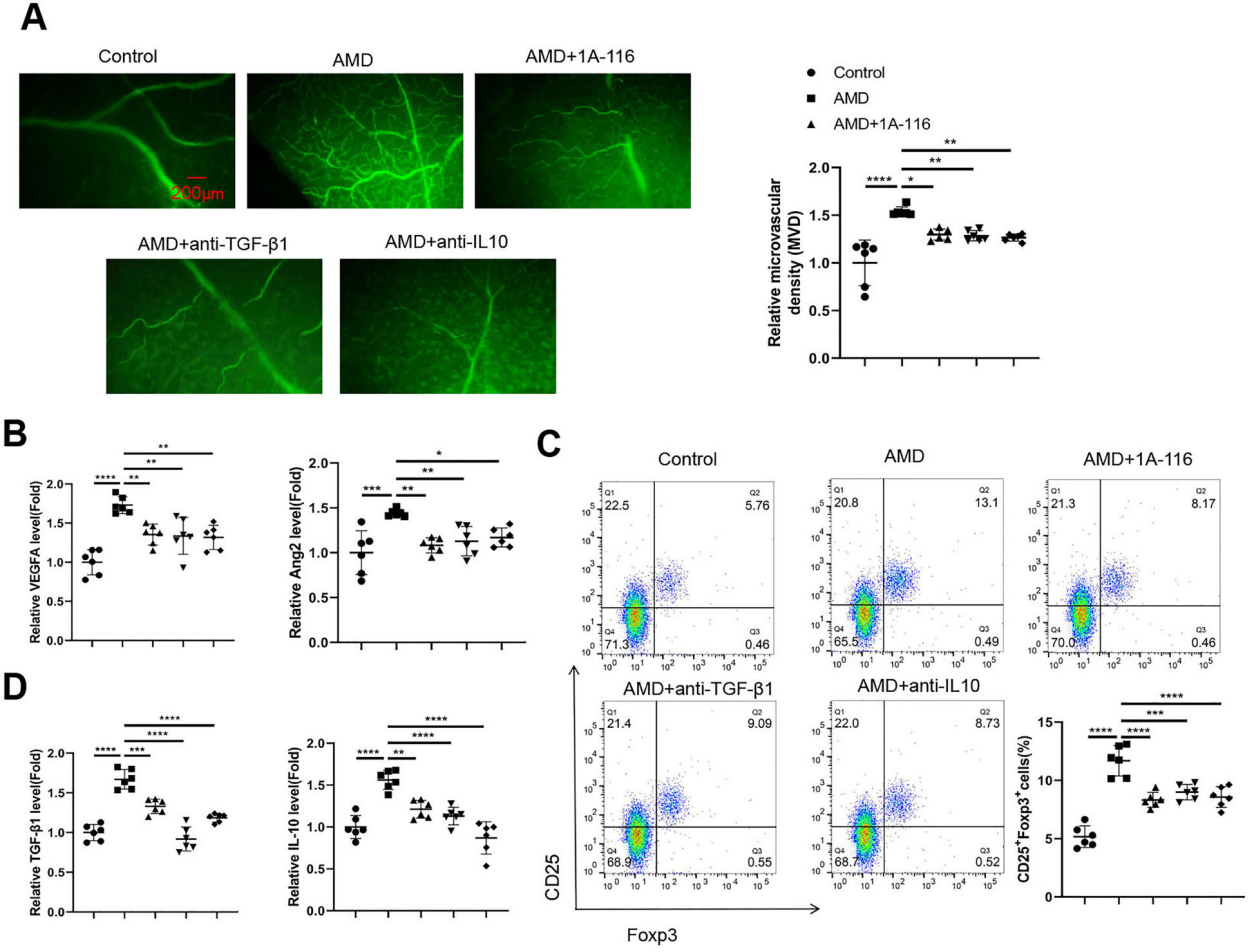
Rac1 activity mediated Treg functions to support choroidal neovascularization in a mouse model of age-related macular degeneration (AMD). A mouse model of AMD was established by laser induction, and either a Rac1 inhibitor (1A-116), a neutralizing antibody against IL-10, or a neutralizing antibody against transforming growth factor (TGF)-β1 was administered for intervention. **A**, Immunofluorescence staining for CD34 in the retina tissues from each experimental group. The microvascular density (MVD) was determined by quantifying the relative CD34+ area in each field (scale bar 200 μm). **B**, The relative levels of the pro-angiogenic factors (VEGFA and Ang2) in ocular venous blood samples were analyzed by ELISA. **C**, Flow cytometry analysis of CD25+FoxP3+ Tregs in the CD4+ T cell population from the blood samples. **D**, ELISA analysis of TGF-β1 and interleukin (IL)-10 levels in the blood samples from each experimental group. Data are reported as means±SD; n=6 animals per group, and 6 independent samples per group were analyzed for each experiment. *P<0.05; **P<0.01; ***P<0.001; ****P<0.0001 (ANOVA).

We next examined the role of Rac1 activity in Tregs and its implication in choroidal neovascularization in the laser-induced AMD mouse model. Immunohistochemistry staining of CD34 in the retina tissues showed that after laser induction in the AMD model group, there was an increased MVD compared to the control group. The application of a Rac1 inhibitor (1A-116) or neutralizing antibodies against TGF-β1 or IL-10 suppressed choroidal neovascularization in the AMD model, respectively (Figure 6A, P<0.05, P<0.01, and P<0.0001). The application of Rac1 inhibitor or neutralizing antibodies against TGF-β1 or IL-10 also reduced the levels of the pro-angiogenic factors (VEGFA and Ang2) in ocular venous blood samples of AMD mice (Figure 6B, P<0.05, P<0.01, P<0.001, and P<0.0001). In the blood samples of the AMD group, there was a significant increase in the percentage of CD25+FoxP3+ Tregs within the CD4+ T cell population, while the administration of the Rac1 inhibitor or neutralizing antibodies against TGF-β1 or IL-10 reduced the Treg percentage in the AMD mice (Figure 6C, P<0.001 and P<0.0001). The reduction of Treg proportion was accompanied by the attenuation of TGF-β1 and IL-10 levels in the blood samples of AMD mice administered with the Rac1 inhibitor or neutralizing antibodies against TGF-β1 or IL-10 (Figure 6D, P<0.01, P<0.001, and P<0.0001). Collectively, our data indicated that the enhanced Rac1 activity in Tregs may contribute to the augmented production of TGF-β1 or IL-10 to promote choroidal neovascularization in the mouse model of AMD.

## Discussion

This study aimed to investigate the role of Tregs and the Rac1 signaling pathway in modulating Treg-derived cytokine expression and their contribution to choroidal neovascularization in wet AMD. Our key findings demonstrated an increased percentage of Tregs in the CD4+ T lymphocyte population of wet AMD patients. Tregs isolated from these patients exhibited upregulated Rac1 expression along with elevated production of the cytokines IL-10 and TGF-β1. Rac1 silencing suppressed Treg stability, impaired Treg differentiation, and attenuated the pro-angiogenic effects of Tregs on human choroidal endothelial cells *in vitro*. Furthermore, administration of a Rac1 inhibitor or neutralizing antibodies against IL-10/TGF-β1 reduced Treg abundance and ameliorated choroidal neovascularization in a mouse model of AMD. Although we cannot exclude the possibility that IL-10 and TGF-β1 derived from other immune cells may also be implicated in AMD progression, our data suggested that Rac1 upregulation in Tregs induced IL-10 and TGF-β1 production to promote choroidal neovascularization in wet AMD. Targeting Rac1 and Treg-derived IL-10/TGF-β1 may ameliorate AMD progression.

Foxp3+ Tregs play an important role in suppressing the autoimmune response and maintaining self-tolerance. Natural Tregs differentiate in the thymus based on their high TCR affinity for autoantigens and migrate to other organs to maintain peripheral immune tolerance. In addition, Tregs derived from extrathymic differentiation can inhibit excessive immune response under inflammatory conditions (22). Nevertheless, the role of Tregs in AMD remains elusive. There is a potential link between Th17/Treg imbalance in aged-related eye diseases, but the link to AMD progression remains to be clarified (23). In experimentally induced uveoretinitis, local retinal Treg depletion aggravated the organ-specific autoimmunity (24), suggesting an essential role of Tregs in maintaining immune homeostasis in the retina. Depletion of Tregs also exacerbated age-associated retinal neurodegeneration (25). Our data add novel evidence that the overpopulation of Tregs may contribute to the choroidal neovascularization in wet AMD. This seems to be consistent with the previous observation that the expression of the Treg master regulator FoxP3 was elevated in the retinal tissues of AMD patients (20). There is also a report showing that the frequency of Th1 cells was lower in patients with wet AMD (26). However, whether the expansion of the Treg population contributes to the reduced frequency of Th1 cells in wet AMD patients warrants further investigation.

Choroidal neovascularization in AMD has been associated with chronic inflammation due to the secretion of pro-inflammatory cytokines such as IFN-γ, IL-17, and IL-1β by Th17 cells and macrophages (27-30). For example, macrophages are preferentially polarized into the M2-like pro-angiogenic phenotype in both the murine model of AMD and in the aqueous humor of AMD patients (28). The inhibition of IL-1β blocked choroidal neovascularization and protected against retinal neurodegeneration (11). We further showed that Treg-derived immunosuppressive cytokines such as IL-10 and TGF-β1 were elevated in blood samples of wet AMD patients, which could be attributed to the increased Treg abundance and their enhanced ability to produce these cytokines. Consistent with our data, there is also evidence showing the increased expression of TGF-β1 in the vitreous humor samples of AMD patients (31,32). We further showed that in the animal model of laser-induced AMD, both TGF-β1 and IL-10 levels were significantly increased in the ocular venous blood samples. These findings were in agreement with a previous study using the same mouse model of AMD (19). Furthermore, previous studies have demonstrated that TGF-β signaling inhibition could ameliorate choroidal neovascularization in the animal model of AMD (33,34). Under hypoxia, increased expression of IL-10 in retinal tissue can stimulate the production of VEGF from macrophages to boost retinal angiogenesis (18,35). Loss of IL-10 in hematopoietic cell lineages caused a significant reduction in choroidal neovascularization upon optical laser burning in the mouse model (36). We also showed that *in vivo* neutralization of TGF-β1 and IL-10 could limit choroidal neovascularization in the mouse model of AMD. Thus, our data and previous evidence indicate that targeting TGF-β1 and IL-10 could be employed as a potential strategy to ameliorate AMD progression.

Rac1 belongs to the RAS family of small GTPase, which regulates the cytoskeleton organization, cell division, and the activation of protein kinases (37). There is increasing evidence that small GTPases are implicated in the development and functions of different T lymphocyte subsets (38). Intriguingly, we showed that Rac1 was overexpressed in Tregs from AMD patients and in the mouse model of AMD. Silencing Rac1 dampened FoxP3 expression and impaired the ability to produce TGF-β1 and IL-10 in Tregs. These data suggested that Rac1 was a key factor for maintaining Treg stability and enhancing the ability to produce TGF-β1 and IL-10. The overexpression of Rac1 in Tregs may be related to the elevated stimulation of pro-inflammatory cytokines in AMD conditions, since pro-inflammatory factors have been shown to trigger the overexpression or activation of Rac1 in other pathogenic conditions (39). The deletion of Rac1 in the myeloid lineage largely abolished the production of inflammatory cytokines in the mouse model of renal injury (40). Nevertheless, the mechanisms by which Rac1 strengthens the functional stability of Tregs warrant future investigations.

Although our study provides valuable insights into the role of Tregs and Rac1 in wet AMD, we acknowledge the inherent limitations associated with studying human samples and the potential confounding factors that may influence our findings. One limitation of our study was the use of peripheral blood samples from patients with wet AMD, dry AMD, and healthy controls to examine Treg profiles and Rac1 expression levels. Circulating Treg and Rac1 levels in the blood may not accurately reflect the pathological changes occurring within the eye tissues. Additionally, certain treatments or medications taken by the patients may have influenced the expression levels of Tregs and Rac1, potentially confounding our findings. While we have verified our results in animal models of choroidal neovascularization, future studies directly examining ocular tissues could provide further insight into the role of Tregs and Rac1 in wet AMD pathogenesis. Furthermore, our study was limited in its ability to explore potential associations between changes in Treg and Rac1 levels with specific clinical features or disease manifestations due to the sample size and patient variability. Further studies involving larger patient cohorts and more advanced techniques for studying ocular tissues may help to further elucidate the mechanisms underlying wet AMD and the roles of Tregs and Rac1 in this disease process. While the current study lays the groundwork, future large-scale longitudinal studies are necessary to comprehensively map the dynamic involvement of Tregs and Rac1 throughout the different phases of wet AMD development. Such analyses could reveal critical windows where these factors exert maximal influence, potentially guiding the timing of therapeutic interventions. Finally, the precise mechanism by which Rac1 controls Treg function needs to be further scrutinized using omics approaches such as transcriptomics and epigenomics.

## Conclusions

In conclusion, our key findings demonstrated that Rac1 upregulation in Tregs promoted IL-10 and TGF-β1 production to mediate choroidal neovascularization in wet AMD. Targeting the Rac1 signaling axis and modulating Treg-derived cytokines may serve as a potential therapeutic strategy for ameliorating AMD progression. Future studies elucidating the precise mechanisms by which Rac1 controls Treg function and employing omics approaches are warranted to uncover novel therapeutic targets.
